# Anaphylatoxins spark the flame in early autoimmunity

**DOI:** 10.3389/fimmu.2022.958392

**Published:** 2022-07-25

**Authors:** Jovan Schanzenbacher, Jörg Köhl, Christian M. Karsten

**Affiliations:** ^1^ Institute for Systemic Inflammation Research, University of Lübeck, Lübeck, Germany; ^2^ Division of Immunobiology, Cincinnati Childrens Hospital Medical Center, University of Cincinnati College of Medicine, Cincinnati, OH, United States

**Keywords:** complement, anaphylatoxins, C3a, C5a, early autoimmunity, break of tolerance

## Abstract

The complement system (CS) is an ancient and highly conserved part of the innate immune system with important functions in immune defense. The multiple fragments bind to specific receptors on innate and adaptive immune cells, the activation of which translates the initial humoral innate immune response (IR) into cellular innate and adaptive immunity. Dysregulation of the CS has been associated with the development of several autoimmune disorders such as systemic lupus erythematosus (SLE), rheumatoid arthritis (RA), ANCA-associated vasculitis, and autoimmune bullous dermatoses (AIBDs), where complement drives the inflammatory response in the effector phase. The role of the CS in autoimmunity is complex. On the one hand, complement deficiencies were identified as risk factors to develop autoimmune disorders. On the other hand, activation of complement can drive autoimmune responses. The anaphylatoxins C3a and C5a are potent mediators and regulators of inflammation during the effector phase of autoimmunity through engagement of specific anaphylatoxin receptors, i.e., C3aR, C5aR1, and C5aR2 either on or in immune cells. In addition to their role in innate IRs, anaphylatoxins regulate humoral and cellular adaptive IRs including B-cell and T-cell activation, differentiation, and survival. They regulate B- and T-lymphocyte responses either directly or indirectly through the activation of anaphylatoxin receptors *via* dendritic cells that modulate lymphocyte function. Here, we will briefly review our current understanding of the complex roles of anaphylatoxins in the regulation of immunologic tolerance and the early events driving autoimmunity and the implications of such regulation for therapeutic approaches that target the CS.

## Introduction

The complement system (CS) is an ancient and highly conserved part of the innate immune response (IR) comprising soluble proteins and membrane-bound receptors bridging innate immunity and adaptive immunity (1). Aside from its well-appreciated canonical activation pathways, non-canonical mechanisms have been recently described, which orchestrate the cleavage and activation of complement factors both in the circulation and intracellularly in immune cells (2). The broad implications of complement activation for health and disease have been reviewed elsewhere (3). Canonical complement activation occurs *via* three different pathways, i.e., the classical pathway (CP), the lectin pathway (LP), and the alternative pathway (AP), all of which converge at the level of C3, eventually resulting in terminal pathway (TP) activation and subsequent membrane attack complex (MAC) formation (4). While the CP and the LP have critical roles in the initiation of the complement cascade and/or pathogen recognition, the AP accounts for amplification and the majority of terminal complement activation (5). In autoantibody-mediated autoimmune diseases, the deposition of immunoglobulin G (IgG) immune complexes can activate the CP (6). During this process, several cleavage products of C3 and C5 are formed that can activate multiple cells of the immune system *via* their corresponding complement receptors (7–9). The two cleavage fragments C3a and C5a, the so-called “anaphylatoxins”, significantly contribute to inflammation and the activation of cells through ligation of their cognate anaphylatoxin receptors C3aR, C5aR1, and C5aR2. Anaphylatoxins are potent chemoattractants that recruit several types of phagocytes to the site of inflammation and mobilize reactive oxygen species in macrophages (10), eosinophils (11), and neutrophils (12). Due to their strong pro-inflammatory properties, they significantly contribute to the pathogenesis of many acute and chronic inflammatory diseases (13).

Autoimmune diseases are a group of chronic inflammatory diseases in which a combination of genetic and environmental factors leads to activation of self-reactive lymphocytes that escaped the multiple layers of central and peripheral tolerance (14). The underlying mechanisms leading to the loss of self-tolerance are multifaceted (15). Most autoreactive lymphocytes are removed at two main checkpoints, i.e., the thymus and the bone marrow by central tolerance mechanisms including deletion and editing. After lymphocytes exit the primary lymphoid organs, several mechanisms of peripheral tolerance ensure that many self-reactive lymphocytes, which escaped central tolerance, are removed from the system (16–18). For this complex process to be successful, a tightly regulated interplay of dendritic cells (DCs), CD4^+^ T cells, and B cells is required. Dysregulation can lead to a break of tolerance that initiates and drives the early phase of autoimmunity, followed by the effector phase where innate and adaptive effector cells promote multiple inflammatory responses. The CS is critically involved in the immunopathology of several autoimmune diseases, including systemic lupus erythematosus (SLE), rheumatoid arthritis (RA), and autoimmune bullous dermatoses (AIBDs) such as bullous pemphigoid (BP) and epidermolysis bullosa acquisita (EBA) where it significantly shapes the effector phase of such diseases by recruiting effector cells to the sites of inflammation (19–24).

During the past decade, our understanding of the mechanisms underlying complement-mediated inflammation during the effector phase of several autoimmune diseases has markedly improved. In contrast, we are still at the beginning to delineate the multiple (path)ways by which the CS contributes to the initiation of autoimmunity. Here, we provide an overview of our current understanding and potential future developments in the field.

## The dual role of complement in autoimmunity

The role of complement in autoimmunity is complex. On the one hand, complement activation is associated with the progression of several autoimmune disorders (19, 20, 23, 24). On the other hand, complement can also protect from autoimmunity.

The deficiency of complement factors that drive the activation of the CP such as C1, C2, and C4 is strongly associated with the development of SLE (25). As part of the C1 complex, binding of C1q to IgG or IgM immune complexes results in activation of the CP (26). Roughly 90% of patients with deficiency in C1q develop lupus-like manifestations (27). Recently, an elegant study shed new light on the role of C1q in the development of SLE (28). The authors demonstrated that C1q limits tissue damage by acting as a “metabolic rheostat” for effector CD8^+^ T cells that drive autoimmune inflammation through the generation of autoantigen fragments *via* granzyme B. In contrast to patients with C1q deficiency, only 10%–20% of patients with a C2 deficiency develop lupus (29). The milder disease manifestation in C2-deficient patients might be explained by a C2 bypass mechanism that leads to activation of terminal complement by C1q and mannose-binding lectin (MBL) (30).

While the incidence of SLE among C3 deficiency is very low (19), reports for C4 deficiency differ depending on the ancestral and ethnic background of the patients (31–35). In a study with over 6,000 lupus patients and healthy controls of European ancestry, both C4 isoforms appeared to be protective relative to complete C4 deficiency. However, patients deficient in C4A were at a higher relative risk than patients deficient in C4B (36). When either human C4A or C4B was expressed in a lupus-susceptible strain (37), mice expressing C4A developed less humoral autoimmunity than C4B-expressing mice. This included a decrease in the number of germinal centers (GCs), autoreactive B-2 cells, autoantibodies, and memory B cells, where the higher efficiency of C4A in inducing self-antigen clearance was associated with the follicular exhaustion of autoreactive B-2 cells. In summary, recent findings provided detailed insights into the mechanisms underlying the protective effects of C1q in the context of autoimmunity; however, the picture regarding the protective effects of C2 and C4 is still sketchy and demands further studies.

In contrast to the protective effect of C1q, C2, and C4, C3 cleavage fragments serve as important cofactors to mount a strong humoral IR. An elegant series of experiments from the Carroll lab demonstrated that binding of C3d-opsonized antigens to complement receptor 2 (CR2; CD21) serves as an important mechanism to foster the uptake of immune complexes by naive B-2 cells within the lymphatics and deliver them to follicular DCs (FDCs) in the B-cell compartment. Furthermore, they identified CR2/CD21 as an important coreceptor for the CD19/CD81 complex that augmented B-cell receptor (BCR)-mediated activation through antigen-tagged C3d that links the CD21/CD19/CD81 complex with the BCR. Finally, CR2 is critical to retain antigens on FDCs, which is crucial for the GC reaction and formation of memory B cells (38).

C3 activation initiates the formation of C5 convertases that activate the terminal pathway. This pathway is characterized by the cleavage of C5 into C5a and C5b, the latter of which initiates the formation of the C5b-9 complex that can form pores as the MAC and destroy pathogens. Aside from its beneficial cytolytic effector functions, the MAC also contributes to inflammation and tissue damage and is closely linked to several autoimmune diseases, such as SLE, where its deposition is associated with disease intensity and used as a marker for treatment response (39). The smaller C5 cleavage, C5a, binds to two distinct receptors, i.e., C5aR1 (CD88) that mediates many of the effector functions of C5a and C5aR2 (C5L2; GPR77), which has initially been considered a mere decoy receptor due to its missing coupling to G-proteins. However, more recent findings identified several C5aR2-mediated functions in inflammation and immunity either in concert with C5aR1 or even independent of C5aR1 (40–43). C5a is a crucial player in the effector phase of various autoimmune disorders, where it drives disease progression through the recruitment and activation of neutrophils and macrophages, depending on the disease (44–49).

By binding to its cognate C3aR, the second anaphylatoxin, C3a, adds to the inflammatory response by activation of innate and adaptive immune cells. In addition to its function as a chemoattractant and activator of eosinophils and mast cells, C3a regulates B-cell and T-cell responses (50, 51). Similar to C5a, C3a plays important roles in the effector phase of different autoimmune disorders including SLE and autoimmune encephalitis (52, 53), where C3a/C3aR signaling promotes the infiltration of neutrophils and macrophages/monocytes. Also, elevated levels of C3a have been associated with disease progression in RA and SLE (54, 55). Of note, C3a can also exhibit anti-inflammatory properties such as preventing the mobilization and degranulation of neutrophils in acute inflammation (56). In summary, C3 cleavage fragments promote humoral autoimmune responses, the MAC can facilitate tissue damage in the context of autoimmunity, and the chemoattractant properties of anaphylatoxins orchestrate the effector phase of many autoimmune disorders.

## From the break of tolerance to early autoimmunity

The maintenance of tolerance underlies a complex interplay between DCs, T follicular helper (TFH) cells, and B cells, where dysregulation can lead to a break of tolerance and the development of autoimmunity. Here, we discuss our current understanding of anaphylatoxin receptor signaling as a regulator of early autoimmunity.

DCs are specialized in priming different types of effector T cells and thus possess the unique ability to control both immunity and tolerance. DCs capture antigens at several mucosal surfaces and then migrate to the lymph nodes, where major histocompatibility complex (MHC)-I- or II-loaded peptides are recognized by T cells *via* the T-cell receptor (TCR) (57). Immature DCs can keep tolerance by presenting self-antigens to T cells in the absence of appropriate costimulation. After receiving appropriate stimuli from pattern recognition receptors, they can differentiate into mature DCs (58) that show a reduced endocytic activity associated with a strong upregulation of MHC-II and costimulatory molecule expression (59). These changes enable them to efficiently drive activation of naive T cells and their differentiation into distinct effector T cells through immunomodulatory signals mediated *via* cell-to-cell contacts and the release of a defined set of cytokines such as IL-12, IL-23, and IL-6 (57, 60).

**Figure 1:**
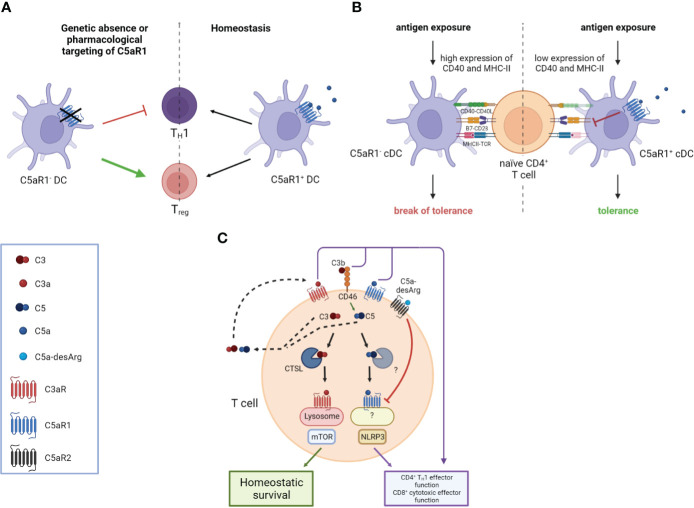
Impact of the anaphylatoxins on DC-mediated and intrinsic T cell activation. **(A)** The genetic absence or pharmacological targeting of C5aR1 on DCs leads to attenuated T helper type 1 (T_H_1) immunity and an increased frequency of Regulatory T cells (Tregs). **(B)** C5aR1 signaling on naïve mucosal conventional DC2 (cDC2) controls the expression of CD40 and MHC-II which determines the threshold of naïve CD4^+^ T cell activation. Mucosal antigen exposure is associated with decreased C5aR1 expression; the lack of C5aR1 expression in cDC2s releases the break on CD40 and MHC-II expression resulting in strong CD4^+^ T cell proliferation and the break of mucosal tolerance. **(C)** T cell activation triggers the secretion of preformed C3 and C5 into the extracellular space, which can be cleaved into C3a, C3b, C5a, and C5b by canonical and non-canonical mechanisms. Binding of these complement fragments to their respective receptors on the T cell induces CD4^+^ T_H_1 and CD8^+^ effector T cell functions. C3 and C5 are also processed intracellularly by proteases such as cathepsin L (CTSL) in the case of C3 and an unknown protease in the case of C5, respectively. Intracellular C3a is critical to maintain low-level mechanistic target of rapamycin (mTOR) activity by binding to C3aR on lysosomes, thereby contributing to the homeostatic survival of CD4^+^ T cells. The cleavage of intracellular C5 into C5a and C5b is enhanced by CD46-mediated signaling. C5a engages C5aR1 triggering NOD-like receptor family pyrin domain containing 3 (NLRP3) inflammasome assembly, eventually driving T_H_1 differentiation of CD4^+^ T cells and CD8^+^ effector T cell functions. Importantly, autocrine engagement of surface-expressed C5aR2 by C5a-desArg can control intracellular C5aR1 activity. Created in BioRender.com.

Activation of C5aR1 on DCs has a strong impact on proliferation and differentiation of naive T cells (48). In anti-neutrophil cytoplasmic antibody (ANCA)-associated vasculitis (AAV), ANCAs produced by autoreactive B-2 cells activate neutrophils, resulting in direct endothelial injury and extensive glomerular deposition of myeloperoxidase (MPO) (61, 62). Here, the response of MPO-specific T cells to glomerular MPO, mediated by C5aR1 on DCs, contributes significantly to necrotizing glomerulonephritis (63, 64). In experimental anti-MPO glomerulonephritis, genetic or pharmacologic C5aR1 targeting resulted in attenuated T_H_1 immunity and increased frequency of regulatory T cells (Tregs) eventually mitigating autoimmunity to MPO (48) (**Figure 1A**).

**Figure 2:**
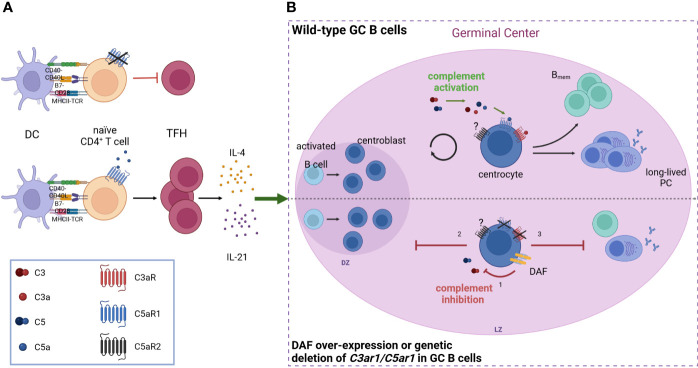
Anaphylatoxin receptor activation on TFH cells and GC B cells controls the production of IgG (auto)antibodies **(A)** C5aR1 signaling on CD4+ T cells facilitates the expansion of TFH cells and their subsequent production of IL-4 and IL-21, which are crucial for the germinal center reaction. The absence of C5aR1 signaling leads to an attenuated TFH cell expansion and reduced GC reaction. **(B)** During the GC reaction, C3a and C5a engage their receptors, C3aR and C5aR1 respectively, on GC B cells, driving B cell proliferation and differentiation into memory B cells (B_mem_) as well as long-lived plasma cells (PC). When complement activation is inhibited (bottom) either by decay-accelerating factor (DAF, CD55) over-expression or deletion of C3aR/C5aR1 on GC B cells, (1) GCs collapse prematurely due to impaired dark zone re-entry and affinity maturation (2), resulting in decreased generation of Bmem and long-lived PCs (3). Created in BioRender.com.

Furthermore, a strong link between complement receptor signaling and the regulation of DCs and TFH cells in the context of immunological tolerance has been described. Activation of the C5/C5a/C5aR1 axis controlled the development of maladaptive T_H_2/T_H_17 development by shifting the balance between immunogenic pulmonary CD11b^+^ conventional DCs (cDCs) and tolerogenic plasmacytoid DCs (pDCs), thereby regulating Th2 cytokine production (65–67). Recently, pulmonary C5aR1^+^ and C5aR1^-^ cDC2 subsets have been described (68), which showed a distinct impact on cDC function after one-time allergen exposure. *Ex vivo* allergen pulsing resulted in low expression of CD40 and MHC-II in the C5aR1^+^ cDC2 subset, leading to minor antigen-specific proliferation of CD4^+^ T cells. In sharp contrast, missing C5aR1 activation either in C5aR1^-^ cDC2s or by C5aR1 targeting induced strong CD4^+^ T-cell proliferation, suggesting that C5aR1 activation on pulmonary cDC2s controls pulmonary tolerance toward aeroallergens by downregulation of CD40 (**Figure 1B**). Furthermore, several studies found C5aR1 activation on T cells as a key mechanism to control T_H_1 differentiation both in mice and man (69–73). For example, in a model of lupus-like chronic graft-versus-host disease (GvHD), genetic or pharmacological ablation of C5aR1 in CD4^+^ T cells protected from the generation and expansion of TFH cells, GC B cells, and autoantibodies (74). Furthermore, C5aR1 antagonism initiated in mice with established bronchiolitis obliterans syndrome ameliorated disease manifestation and reduced the associated differentiation of TFH and GC B cells. These findings emphasize the critical role of C5aR1 in supporting TFH cell differentiation and its subsequent impact on the GC reaction and (auto)antibody production (**Figure 2A**).

In addition to its impact on DC and TFH functions, anaphylatoxin receptor activation also regulates the function of Tregs. Natural CD4^+^ FoxP3^+^ Tregs (nTregs) are crucial for immune homeostasis, the persistence of self-tolerance, and hence underlie strict control mechanisms to ensure protective immunity (75–77). Based on findings showing that the activation, differentiation, and expansion of conventional CD4^+^ CD25 T cells are linked to C3aR and C5aR1 signaling (69, 78–80), Kwan et al. (81) investigated the modulation of nTreg functions by C3a and C5a. They found that C3aR and C5aR1 activation on nTregs inhibited their function by inducing phosphorylation of the transcription factor Foxo1, resulting in reduced FoxP3 expression on nTregs.

More recent work by Liszewski et al. (82) unraveled a novel and unexpected role for an intracellular CS, which they termed the “complosome,” that regulates key metabolic pathways critical for the survival of peripheral human T cells and their effector functions. They showed that circulating human CD4^+^ and CD8^+^ T cells continuously generate low levels of C3a and C3b by cathepsin L-mediated cleavage of intracellular C3, resulting in mammalian target of rapamycin (mTOR) activation *via* lysosome-bound C3aR engagement and interaction of complosome C3b with surface-bound CD46. These mechanisms were shown to be crucial for T-cell homeostasis, CD4^+^ T_H_1 effector function, and CD8^+^ cytotoxic T-cell effector activity (83). This interesting finding implicates that human T_H_1 and T_H_17 responses are regulated by autocrine and intracellular complement activation, shedding new light on the role of complement in controlling immunological tolerance (**Figure 1C**).

In addition to its impact on cellular immunity, anaphylatoxins also regulate humoral IR. Effective humoral IRs rely on high-affinity antibodies generated by affinity maturation in the GCs within the secondary lymphoid organs (84, 85). Here, B-2 cells go through repeated cycles of somatic hypermutation, clonal expansion, and affinity-governed positive selection. Positive selection is orchestrated by costimulatory signals from TFH cells that have been recruited to the GCs after antigen capture. Depending on these signals, non-self-reactive GC B cells survive and proliferate, whereas self-reactive GC B cells undergo either further differentiation or cell death (85–88). In this stringently regulated process, mTOR signaling and expression of the proto-oncogene c-MYC exert crucial functions (89, 90). In a recent study, Cumpelik et al. (91) found downregulation of the complement inhibitor decay-accelerating factor (DAF, CD55) in GC B cells *via* B-cell lymphoma 6 (Bcl-6) associated with simultaneously increased expression of MAC inhibitor CD59. The reduced complement regulation resulted in increased C3 and C5 cleavage on GC B cells leading to increased generation of C3a and C5a and consecutive enhanced C3aR and C5aR1 signaling. Importantly, this process was indispensable for positive selection and GC function, as disruption of this pathway decreased mTOR activity in response to BCR-CD40 signaling, eventually leading to a premature GC collapse and defective affinity maturation (**Figure 2B**).

Furthermore, combined C3aR and C5aR1 signaling was shown to modulate antibody production and class switch recombination of B-2 cells (92). Using *C3aR^-/-^C5aR1^-/-^* mice, Paiano et al. found that C3aR/C5aR1 signal transduction was indispensable for CD40 upregulation, IL-6 production, proliferation, and IL-21 production by follicular CD4^+^ T cells. Furthermore, using immunized mice deficient in systemic C3 and C5 and transfecting them with wild-type bone marrow (BM), the study showed that locally produced complement was necessary for this signaling pathway and sufficient for the initial B-2 antibody response.

In addition to B-2 cells that generate high-affinity antibodies against foreign antigens, B-1 cells mediate the first line of immune defense through low-affinity natural IgM antibodies. Interestingly, B-1 cells have also been shown to drive the establishment of autoimmune-mediated diseases, such as type 1 diabetes (93) and SLE (94). Their regulation is also highly dependent on C5a (95), as the C5a/C5aR1 axis controls the trafficking of B-1 cells into the BM, the peritoneal cavity, and from the BM to the spleen, emphasizing the importance of the C5a/C5aR1 axis in early autoimmunity.

Based on a growing body of evidence showing that complement not only controls the effector phase of many autoimmune disorders but the early events of humoral and cellular adaptive immune responses, complement pathways and mediators have sparked the interest as therapeutic targets to treat autoimmune disorders (96). At this point, only a few complement inhibitors have been approved for therapeutic use, including the C5 inhibitor eculizumab, the plasma C1 protease inhibitor (C1INH), the C3 inhibitor pegcetacoplan, and the C5aR1 antagonist avacopan. While eculizumab treatment has been approved for the treatment of paroxysmal nocturnal hemoglobinuria (PNH), atypical hemolytic uremic syndrome (aHUS), and neuromyelitis optica spectrum disorders (NMOSDs), C1INH is used for the treatment of hereditary angioedema (96). Pegcetacoplan is currently approved for the treatment of PNH (97) and the first C5aR1 inhibitor, avacopan, for the treatment of AAV (98).

## Conclusion

Apart from the well-known functions of recruiting and activating innate effector cells that drive the pro-inflammatory environment of many autoimmune diseases, anaphylatoxin receptor signaling appears to also ignite the early events of humoral and adaptive immunity, leading to the loss of tolerance as a first step to induce autoimmunity. Exemplarily, local generation of C3a and C5a in tissues or inside DCs, T cells, and B cells and activation of their cognate anaphylatoxin receptors in an autocrine or paracrine fashion function as one important rheostat to keep tissue homeostasis and immunologic tolerance. The growing understanding of the multiple facets of anaphylatoxin functions opens new perspectives for spatially and temporarily tailored targeting strategies that consider the interindividual differences in immune responses. A few drugs are already Food and Drug Administration (FDA)-approved that target the complement system at the level of C3, C5 or more specifically inhibit C5a-mediated C5aR1 activation. The multiple complement inhibitors that are currently tested in clinical trials open up a wide range of new treatment options for clinicians to inhibit the distinct activation pathways or complement fragment-mediated activation of specific complement receptors (99, 100). However, the already approved drugs and the compounds tested in ongoing clinical trials are designed for the treatment of acute inflammation. In light of the impact on humoral and cellular adaptive immune responses of C3 and C5 cleavage fragments, it will be of major importance in future studies to define endpoints in clinical studies of autoimmune diseases that also take these crucial functions of complement mediator molecules into account. Also, it will be crucial to discriminate between intracellular and extracellular complement and complement receptor targeting given the major impact of complosome activation for T-cell activation in particular (101).

## Open questions

- How can we translate our findings that anaphylatoxins shape early autoimmunity into appropriate therapeutic approaches?
- When do we target the CS?
- Where do we target the CS, i.e., extracellular complement vs. intracellular complement?
- What is the contribution of C5aR2 to early autoimmunity?

## Author contributions

JS wrote the first draft of the manuscript. JK, CK, and JS wrote sections of the manuscript. All authors contributed to manuscript revision, read, and approved the submitted version.

## Funding

This work was funded by the Research Training Group 2633 Autoimmune Pre-Disease project A8 to CK and JK.

## Conflict of interest

The authors declare that the research was conducted in the absence of any commercial or financial relationships that could be construed as a potential conflict of interest.

## Publisher’s note

All claims expressed in this article are solely those of the authors and do not necessarily represent those of their affiliated organizations, or those of the publisher, the editors and the reviewers. Any product that may be evaluated in this article, or claim that may be made by its manufacturer, is not guaranteed or endorsed by the publisher.
